# Impact of the Lab-Score on Antibiotic Prescription Rate in Children with Fever without Source: A Randomized Controlled Trial

**DOI:** 10.1371/journal.pone.0115061

**Published:** 2014-12-11

**Authors:** Laurence Lacroix, Sergio Manzano, Lynda Vandertuin, Florence Hugon, Annick Galetto-Lacour, Alain Gervaix

**Affiliations:** Pediatric Emergency Medicine Department, Child and Adolescent Medicine, Geneva University Hospital, Geneva, Switzerland; Fondazione IRCCS Ca' Granda Ospedale Maggiore Policlinico, UniversitÃ degli Studi di Milano, Italy

## Abstract

**Background:**

The Lab-score, based on the combined determination of procalcitonin, C-reactive protein and urinary dipstick results, has been shown accurate in detecting serious bacterial infections (SBI) in children with fever without source (FWS) on retrospective cohorts. We aimed to prospectively assess the utility of the Lab-score in safely decreasing antibiotic prescriptions in children with FWS and to determine its diagnostic characteristics compared to common SBI biomarkers.

**Methods:**

Randomized controlled trial in children 7 days to 36 months old with FWS, allocated either to the Lab-score group (Lab-score reported, blinded WBC count) or to the control group (WBC, bands and C-reactive protein determined, blinded procalcitonin and Lab-score), followed up until recovery. Demographic data, antibiotic prescription rate, admission rate and diagnostic properties of the Lab-score were analyzed.

**Results:**

271 children were analyzed. No statistically significant difference concerning antibiotic prescription rate was observed: 41.2% (54 of 131) in the Lab-score group and 42.1% (59 of 140) in the control group (p = 1.000). If recommendations based on the Lab-score had been strictly applied, a hypothetical 30.6% treatment rate would have been encountered, compared to the overall 41.7% observed rate (p = 0.0095). A Lab-score ≥3 showed the following characteristics: sensitivity 85.1% (95% CI: 76.5–93.6%), specificity 87.3% (95% CI: 82.7–91.8%), positive predictive value 68.7% (95% CI: 58.7–78.7%), negative predictive value 94.1% (95% CI: 91.5–97.9%), positive and negative likelihood ratios: 6.68 and 0.17 respectively. Area under the receiver operating characteristic curve was best for the Lab-score (0.911, 95% CI: 0.871–0.950).

**Discussion:**

No difference regarding antibiotic treatment rate was observed when using the Lab-score, due to lack of adherence to the related recommendations. However, if strictly followed, a significant 26.5% reduction of antibiotic prescriptions would have been encountered. Medical education needs to be reinforced in order to observe rather than treat low-risk well-appearing children with FWS.

**Trial Registration:**

ClinicalTrials.gov NCT02179398

## Introduction

Fever without a source is a frequent diagnostic challenge in children presenting to the Pediatric Emergency Department (PED), accounting for approximately 20% of all febrile patients [1], and up to 20% of visits among children in the 2- to 24-month age group across all years [2]. Among these, it is crucial to differentiate those suffering from serious bacterial infections (SBI) and necessitating immediate antibiotic treatment from those presenting with focal bacterial infections or viral infections. SBI include sepsis, occult bacteremia, bacterial meningitis, febrile urinary tract infection (UTI), pneumonia, bacterial enteritis, osteomyelitis and septic arthritis.

Isolated clinical signs, especially general appearance, raised temperature, no fluid intake in the previous 24 hours and increased capillary refill time have been shown the strongest diagnostic markers for SBI in a large prospective cohort of young children presenting with a febrile illness to an emergency department in Australia [3]. Many clinical scores compelling various symptoms and signs, such as the observation scale published by McCarthy et al. in 1982 have been described to detect patients at risk for SBI, however their diagnostic performances are poor [4], [5]. Many algorithms and pathways adding laboratory data to isolated clinical signs have been described for the stratification of SBI risks in the management of febrile infants. Among them, in 1993, Baraff et al. published an algorithm based on the detection of low-risk previously healthy infants and children with FWS. Low-risk criteria for SBI were defined through a combination of clinical criteria (nontoxic clinical appearance) and laboratory criteria (white blood cell (WBC) 5–15’000/mm3, <1’500 bands/mm3, normal urinalysis and <5 WBCs/hpf in stools in case diarrhea was present) [6]. Although these recommendations have been revised over time [1], [7], poor accuracy of clinical signs and WBC and/or band counts to adequately predict SBI accounted for the need to rely on additional diagnostic tools [8]–[10]. CRP and more recently PCT have been shown accurate markers for SBI prediction [11]–[15]. However, isolated biological markers as CRP or PCT also lack sensitivity and/or specificity when analyzed independently, often leading to overprescription of antibiotics. A recent study in 15’781 children less than 5 years presenting with a febrile illness stated that 20% of patients without any SBI nor clinically diagnosed infection were prescribed antibiotics [3].

The recently described and easy-to-perform Lab-score takes into account biological variables independently associated with SBI, weighed differently according to the odds ratio in the univariate analysis in the original derivation study [16]. Table 1 details the contributing elements and their corresponding value according to the best cut-off points. Based on the combined determination of procalcitonin (PCT), C-reactive protein (CRP) and urinary dipstick (UD) results, the Lab-score value consequently ranges from 0 to 9. A cutoff point ≥3 was identified as the best Lab-score value for SBI prediction in the derivation set, with 94% sensitivity (95% CI 74–90) and 78% specificity (95% CI 64–87) in the validation set in the original study including in children aged 7 days up to 36 months of age with FWS [16]. When applied to a large external cohort of children in the same age range with a 22.7% SBI prevalence (pre-test probability), a Lab-score ≥3 showed a good sensitivity of 86% (95% CI 77–92), a good specificity of 83% (95% CI 79–87), a positive likelihood ratio of 5.1 (95% CI 3.9–6.6) and an excellent negative likelihood ratio of 0.17 (95% CI 0.10–0.28) for SBI detection [17].

**Table 1:** Lab-score calculation.

Test	PCT (ng/mL)			CRP (mg/L)			Urinary dipstick	
**Value**	<0.5	0.5–1.99	≥2	<40	40–99	≥100	negative	positive*
**Points**	0	2	4	0	2	4	0	1

*Table adapted from Lacour, A.G., S.A. Zamora, and A. Gervaix, A score identifying serious bacterial infections in children with fever without source. Pediatr Infect Dis J, 2008. **27**(7): p. 654–6.*

*positive leukocyte esterase and/or positive nitrate.

Previous studies concentrated on the diagnostic characteristics of the Lab-score. However, its impact on patient management has never been tested prospectively. The utility of such a diagnostic tool being a more accurate SBI detection, using the Lab-score should induce a reduction of inappropriate antibiotic treatments. The aim of the present study was to assess the usefulness of the Lab-score in safely decreasing unnecessary antibiotics prescription in children with FWS. The second objective of the study was to establish diagnostic characteristics of the Lab-score on a prospective cohort of children with FWS.

## Materials and Methods

The protocol for this trial and supporting CONSORT checklist are available as supporting information; see S1 Checklist and S1 Protocol (English) and S2 Protocol (French).

We performed a randomized controlled trial in children 7 days to 3 years old with FWS, presenting to the PED of a tertiary care urban pediatric center with 25’000 annual visits, between August 1^st^, 2010 and June 30^th^, 2013. FWS was defined by the presence of body temperature 38.0°C (100.4°F) or more, with no identified source of infection after a careful history and a thorough physical examination. Body temperature should have been measured by the parents, caregivers or the nurse at PED presentation, using either rectal, axillary or transtympanic devices. Exclusion criteria were presence of underlying congenital or acquired immunodeficiency syndromes, previous antibiotic administration within 48 hours of presentation and fever for more than 7 days at presentation.

After parental written informed consent was obtained on the day of presentation, eligible children were recruited by residents and randomly assigned to one of the two following groups: the Lab-score group or the control group. In the Lab-score group, a Lab-score ≥3 was used as the sole SBI marker. WBC count with differential were both performed but blinded to the physicians in charge of the patient. In the control group, patients were managed according to the commonly admitted biomarkers for SBI: WBC count >15’000/mm^3^, band count >1’500/mm^3^ and CRP≥40 mg/L. In this group, PCT and the corresponding Lab-score were both performed but remained blinded to the team in charge of the patient. Assignment to either group was achieved using sealed and chronologically numbered envelopes containing indications for one of the previously mentioned group. Randomization was achieved using an Excel-generated random numbers table.

PCT was measured quantitatively using the VIDAS B.R.A.H.M.S PCT assay (bioMérieux, Marcy L'Etoile, France), a one-step automated heterogeneous sandwich immunoassay with fluorescence detection based on anti-procalcitonin antibodies [18]. Total assay time of the VIDAS method is 20 minutes with a measuring range of 0.05 to 200 µg/L and a functional sensitivity of 0.09 µg/L. At a PCT level of 0.22 µg/L, the intra- and inter-assay variations of the VIDAS are 4.6% and 7.0%, respectively (manufacturer's package insert). CRP was determined with the NycoCard CRP system, an immunochemical assay for the quantitative determination of CRP in whole blood, serum and plasma (manufacturer's package insert).

Physicians participating in the study had not been trained to using the Lab-score before. However, they had received the same medical education concerning serious bacterial infection risks and management in infants and children. Oral and written information on the Lab-score was provided together with the information concerning the study. Moreover, indications as to whether antibiotics were recommended or not were offered to both groups according to laboratory findings, but the final decision to initiate treatment was left to the physician in charge of the patient. Even though heterogeneity in participating physicians could lessen the effect of the Lab-score on antibiotic prescription rates, this parameter was still important to be maintained in order to achieve conditions closer to reality and to avoid selection bias. Thirty different physicians participated to the study.

General data were recorded, such as age at the time of the consultation, gender, highest recorded temperature and fever duration before PED presentation. Toxic appearance (defined as lethargy, poor peripheral perfusion, cyanosis, hypo- or hyperventilation) was also noted.

The following laboratory data were analyzed in both groups (even though partially blinded depending on the assigned group): WBC count, band count, CRP, PCT, UD results, Lab-score value, blood culture and urine culture (obtained through urethral catheterization in not already toilet-trained children or with a clean catch sample after thorough disinfection in continent children). Any other investigation aiming at better defining the precise diagnosis (lumbar puncture, chest X-ray, rapid-antigen testing, etc.) could be ordered depending on the clinical presentation of the patient, but were left to the decision of the physician in charge of the patient. The decision for immediate antibiotic prescription was recorded as the primary outcome. Hospital admission rate and the presence of SBI related to the final diagnosis were assessed as secondary outcomes. For this matter, after a 48- to 72-hours delay, a telephone follow-up was carried out by one of the study investigators to assess the evolution of the clinical condition in the affected child, focusing on symptoms or signs that could have appeared in the interval from presentation. When the final diagnosis remained uncertain or when the fever was still present at the time of the initial phone follow-up, a free medical visit was offered to parents and further follow-up calls or visits were organized until definitive resolution of the fever episode for more than 24 hours. The need for secondary antibiotic prescription after the interval period was recorded. When the fever had resolved, one of the senior investigators concluded upon the final medical diagnosis based on the combined determination of clinical, laboratory and other ancillary data, and follow-up.

### Diagnosis of Serious Bacterial Infection

SBI was defined as isolation of a bacterial pathogen from the blood, the urine, the cerebrospinal fluid (CSF), the synovial fluid, the bone, the stools, or by presence of pneumonia defined as presence of fever (≥38°C or ≥100.4°F), cough, tachypnea, and a radiographic lung infiltrate. Urinary tract infection (UTI) was defined as presence of both pyuria (positive leukocyte esterase or positive nitrite test on a urinary dipstick or presence of white blood cells (WBCs) and bacteria on urine microscopic examination) and growth of at least 50’000 colony-forming units (cfu) per mL of a uropathogenic organism cultured from a urine specimen obtained through catheterization, as recommended by the AAP (Subcommittee on Urinary Tract Infection and Steering Committee on Quality Improvement and Management) [19]. Results between 10’000 and 100’000 cfu/mL were evaluated in context, such as whether the urinalysis findings support the diagnostic of UTI and whether the organism is a recognized uropathogen, as suggested by the above-mentioned guidelines. Indeed, frequent bladder emptying in children less than 3 years of age can lead to a lack of urinary nitrate or WBC detection and to a reduced number of bacteria/mL in the fresh urine sample. Therefore, possible urinary tract infection was defined in case of a strong clinical suspicion of UTI when growth of more than 50’000 cfu/mL was present with a negative UD or when facing growth of more than 10’000 cfu/mL of a single or double uropathogen with a positive UD in an appropriately collected specimen of urine. These patients were diagnosed as possible SBI patients and therefore classified in the SBI group (worst case scenario). Positive blood and CSF cultures were defined as culture leading to the isolation of bacteria characterized as true pathogens.

The total number of patients admitted during the study period as well as the number of infants younger than 3 years of age presenting to the PED with FWS during the study period were recorded. The study was approved by the Institutional Ethics Committee (Commission Cantonale d'Ethique de la Recherche, CCER), adheres to the CONSORT 2010 Statement, and is registered on ClinicalTrials.gov (NCT02179398), where data underlying the findings in the study are freely available.

### Statistical Analysis

Anonymized data were recorded using Microsoft Excel Database and then analyzed under PASW 18.0. The adequacy of randomization was tested by comparing both groups. Normally distributed data were expressed as mean ± standard deviation (SD), non-normally distributed data as median and interquartile range (IQR) and categorical data as percentages. Normally distributed data were compared using independent-samples *t* test and non-normally distributed data using Mann–Whitney *U* test. Categorical data were compared using χ2 test. Parameters displaying p-values <0.05 were considered statistically significant.

The diagnostic performance of the Lab-score and other laboratory markers were analyzed using a receiver operating characteristic analysis. Sensitivity, specificity, positive and negative predictive values and likelihood ratios (LRs) for a Lab-score cutoff point ≥3 were calculated and reported with a 95% confidence interval (CI). In the original derivation study, observed antibiotic treatment rate was 60%. However, if the Lab-score had been strictly followed in this cohort, only 30% patients would have received antibiotics, showing an absolute difference of 30%. We thus planned to observe a slightly lower absolute difference of 20% only between the Lab-score group and the usual approach followed in the Control group. Power calculation suggested 97 patients should be enrolled in each group to give 80% power at the 5% level of significance to detect a 20% difference in antibiotic prescription rate. Taking into account the possibility for lost to follow-up patients or missing or incomplete results, we considered including 140 patients in each group. The trial ended after completion of a sufficient number of patients at the expected timing.

## Results

**Figure 1:**
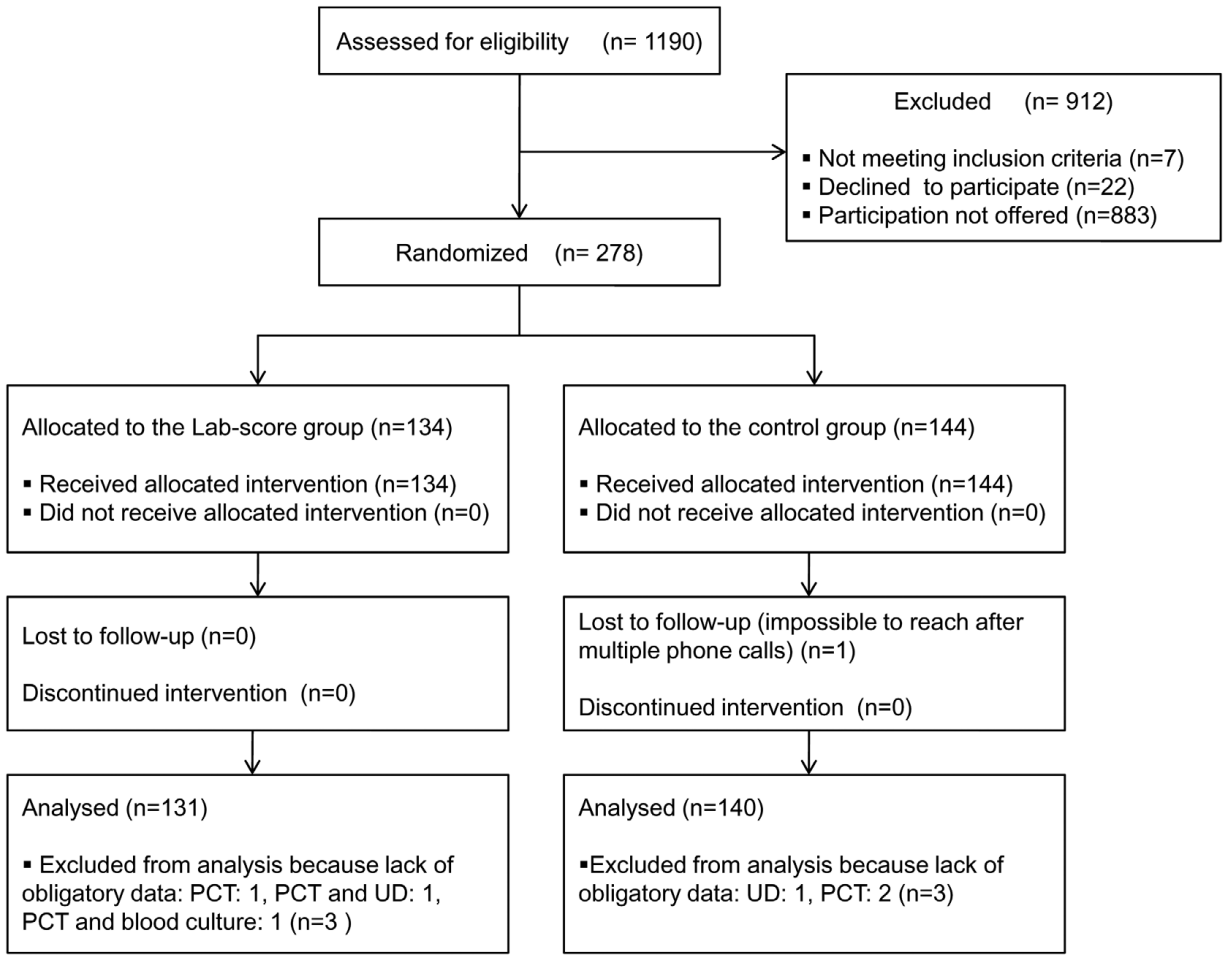
Search results on CONSORT Flow Diagram (Consolidated Standards of Reporting Trials).

A total of 68140 patients attended the PED during the study period (September 1^st^ 2010- June 30^th^ 2013) and 27147 of them were less than 3 years of age. 1190 were diagnosed with fever without source after the PED visit, representing 4.3% of the overall visits in the focus group. 278 patients who met the inclusion criteria were included over the 35 months-study period. However, due to missing obligatory data, 271 children aged 7 days up to 36 months were finally analyzed. Of these, 117 were infants under 3 months of age with FWS. 131 children were assigned to the Lab-score group and 140 to the control group, as shown in Fig. 1.

In the cohort study, 67 patients (24.7%) were diagnosed with SBI or possible SBI (25.6% in the subgroup of children less than 3 months of age), distributed as follows: 51 (76.1%) UTI, 10 (14.9%) possible UTI, 3 (4.5%) pneumonia, 1(1.5%) *Escherichia coli* and enterovirus meningitis, 1 (1.5%) *methicillin-sensitive Staphylococcus aureus* occult bacteremia and 1 (1.5%) *Campylobacter jejuni* enteritis. 193 patients (70.8%) had viral or highly suspected viral infections, and 7 (2.6%) presented with focal bacterial infections on follow-up (5 otitis media, 1 pharingotonsillitis and 1 vulvar cellulitis). Other causes for fever were observed in 3 patients (1.5%): 2 immunization reactions and 1 Kawasaki disease. In a single patient only (0.4%) did the final diagnosis remain unknown due to lack of follow-up.

Table 2 summarizes demographic and baseline characteristics in both groups, showing no statistically significant difference for age, gender, fever duration, maximal measured temperature, Lab-score values, and presence of SBI.

**Table 2:** Baseline demographic and clinical characteristics of patients in the Lab-score group and in the control group.

Variable	Lab-score group n = 131	Control group n = 140	P value
**Age (mo)**	4.8 (1.7–10.4)	3.4 (1.5–10.4)	0.993
**Gender (M/F)**	65/66	71/69	0.953
**Fever duration <12 h 12–24 h>24 h**	47 25 59	55 24 61	0.209
**Maximum temperature (°C)**	39.3±0.8	39.3±0.8	0.710
**Lab-score value**	2.03±2.62	2.04±2.86	0.971
**SBI**	32 (24.4%)	35 (25%)	1.000

Age data are median and interquartile range. Temperature and Lab-score values are expressed as means ± SD.

No statistically significant difference concerning initial antibiotic prescription rate was observed between the two groups: 41.2% (54 of 131) children received antibiotics in the Lab-score group versus 42.1% (59 of 140) in the control Group (p = 1.000). In the subgroup of patients aged less than 3 months, 52.8% (28 of 53) children received antibiotics in the Lab-score group versus 43.8% (28 of 64) in the control group, thereby showing a non significant difference (p = 0.428). Similar findings were found with final antibiotic prescription rates at the time of follow-up.

However, if the Lab-score had been strictly followed in the Lab-score group, a reduction in antibiotic treatment rate would have been observed, although not statistically significant: only 30.5% patients (40 patients of 131) would have been prescribed antibiotics (p = 0.0938).

The Lab-score being also available retrospectively in the control group, we analyzed the effects of strict Lab-score application on the entire cohort of eligible children with FWS (Lab-score and control groups): a very statistically significant theoretical reduction in antibiotic treatment rate would have been observed compared to the observed overall 41.7% (113 of 271) treatment rate. Indeed, only 83 of 271 children (30.6%) would have been treated (p = 0.0095). This difference is even more striking in the subgroup of infants aged less than 3 months: although 52.8% (28 of 53) children in the Lab-score group and 43.8% (28 of 64) in the control group were prescribed antibiotics, only 28.3% (15 of 53) and 23.4% (15 of 64) would have been treated according to the Lab-score recommendations (p = 0.018 and 0.025 respectively).

In the Lab-score group, no statistically significant difference could be raised between the observed antibiotic prescription rate and the hypothetical 44.2% treatment rate if standard recommendations based on WBC and band counts and CRP values had been strictly followed (p = 0.706), no matter the age of the patient. If recommendations to treat all infants with FWS aged less than 28 days old had been strictly applied to the entire cohort, added the previously mentioned WBC count, band count and CRP determination criteria, hypothetical treatment rate would be as high as 54.2% (147 of 271 patients), which represents a statistically significant difference with both the observed treatment rate (p = 0.046) and the hypothetical treatment rate derived from the strict Lab-score application (p<0.0001).

If standard recommendations based on WBC, band count and CRP values had been strictly followed, no matter the age of the patient between 7 days and 3 months of age, 18 (34.0%) of them would have been treated, which represents a non statistically significant difference with the observed prescription rate (p = 0.675). If the recommendation to treat any infant aged less than 1 month with FWS was added to the aforementioned conditions, 33 (62.3%) would have been treated, which is a non statistically significant difference with the observed prescription rate.

On the entire cohort, analysis of the Lab-score values prospectively (for the Lab-score group) and retrospectively (for the control group) showed that 10 patients (3.7%) had a negative Lab-score (<3) but a suspected or proved SBI, including 8 febrile urinary tract infections, 1 possible *methicillin-sensitive Staphylococcus aureus* occult bacteremia and 1 *Escherichia coli* and enterovirus coinfection meningitis. Among these misdiagnosed patients, 6 were aged less than 3 months, and none less than 1 month. The rate of misdiagnosis would have been the same (4.1% patients, 11 out of 271) if guidelines based on the combined determination of WBC and band counts and CRP.

Admission rate showed no difference between the two groups: 33.6% patients (44 of 131) were admitted in the Lab-score group and 35.7% (50 of 140) in the control group (p = 0.810). Similar findings were found in the subgroup of children aged less than 3.0 months, nevertheless with a higher admission rate: 66.0% patients (35 of 53) were admitted in the Lab-score group and 60.9% (39 of 64) in the control group (p = 0.706).

**Figure 2:**
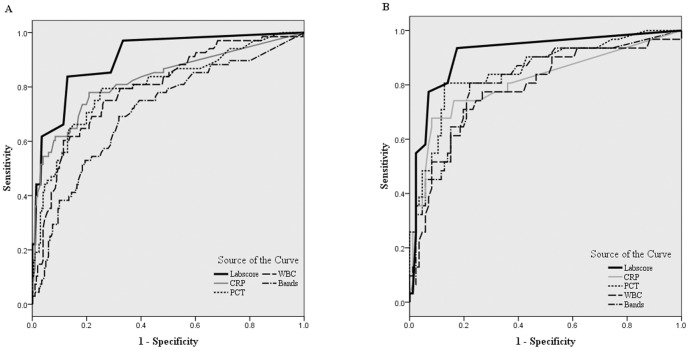
Receiver operating characteristic curve for Lab-score, PCT, CRP, WBC and band counts for SBI detection in children 0-3 years (A) and in children <3 months (B).

Our second objective was to test the Lab-score prospectively. Diagnostic characteristics for SBI detection for a Lab-score ≥3, including sensitivity, specificity, positive and negative predictive values and likelihood ratios are reported in Table 3. Overall, the Lab-score showed a slightly better sensitivity and a much better specificity than WBC≥15’000/mm^3^ or band count ≥1’500/mm^3^ or CRP≥40 mg/L. Areas under the Receiver Operating Characteristic (ROC) curves for Lab-score, PCT, CRP, WBC and band counts are shown in Fig. 2. In the entire cohort of children aged 7 days until 3 years old with FWS, AUC for the Lab-score (0.911, 95% CI 0.871–0.950) was higher than for any other independent single marker concerning SBI detection (CRP: AUC 0.837, 95% CI 0.774–0.899, PCT: AUC 0.816, 95% CI 0.754–0.878, WBC: AUC 0.798, 95% CI 0.735–0.861, bands: AUC 0.720, 95% CI 0.648–0.792). In the subgroup of infants aged less than 3 months old, the Lab-score shows the same superiority (AUC 0.916, 95% CI 0.852–0.981).

**Table 3:** Diagnostic characteristics of the Lab-score and of common biomarkers for the detection of SBI in children.

Parameter	0–3 years (n = 271)		0–3 months (n = 117)	
	Lab-score≥3	WBC≥15’000/mm^3^ or Bands≥1’500/mm^3^ or CRP≥40 mg/L	Lab-score≥3	WBC≥15’000/mm^3^ or Bands≥1’500/mm^3^ or CRP≥40 mg/L
**Sensitivity(95% CI)**	85.1 (76.5–93.6)	83.6 (74.7–92.5)	80.0 (65.7–94.3)	70.0 (53.6–86.4)
**Specificity(95% CI)**	87.3 (82.7–91.8)	68.8 (62.4–75.2)	93.1 (87.8–98.4)	79.3 (70.8–87.8)
**PPV % (95% CI)**	68.7 (58.7–78.7)	47.1 (38.1–56.0)	80.0 (65.7–94.3)	53.8 (38.2–69.5)
**NPV % (95% CI)**	94.1 (91.5–97.9)	92.7 (88.5–96.8)	93.1 (87.8–98.4)	88.5 (81.4–95.6)
**LR +**	6.68	2.68	11.60	3.38
**LR −**	0.17	0.24	0.21	0.38

## Discussion

The management of an infant or a child with FWS often remains challenging although risks for SBI have decreased seriously since the introduction of widespread *Haemophilus* type B and pneumococcal immunization. Various approaches have been proposed regarding SBI detection so far. Among them, the Lab-score has been shown to be an interesting diagnostic tool in retrospective studies to detect children with SBI. The present study is the first to evaluate the Lab-score prospectively, regarding both its impact on antibiotic prescription rates and the diagnostic performance of the test.

Although the Lab-score showed very good diagnostic characteristics regarding SBI detection in children, its application on a prospective cohort of children with FWS could not show any reduction in antibiotic prescription rate. However, if the Lab-score had been strictly followed in the entire cohort, a statistically significant difference would have been encountered: only 30.6% of children would have been treated, which is close to the observed 24.7% SBI or possible SBI rate in our cohort. This would represent a 26.5% reduction in the overall antibiotic prescription rate compared to the hypothetical treatment rate based on the more “classical” and commonly admitted recommendations based on WBC≥15’000/mm^3^ and/or bands ≥1’500/mm^3^ and/or CRP ≥40 mg/L. If we consider that all febrile children less than 28 days must be treated as stated in the actual recommendations, this reduction in prescription rate would be as high as 43.5%. Moreover, using the Lab-score did not permit any statistically significant reduction in admission rate in the Lab-score group compared to the control group.

The proportion of misdiagnosed patients did not differ no matter which of the SBI detection method was used (i.e. Lab-score alone or WBC, band count and CRP). If recommendations concerning the Lab-score had been strictly followed, 3.7% (10 of 271) patients would have been misdiagnosed as probable non-SBI patients. The majority of them (8 patients) suffered from febrile urinary tract infection, but none of them underwent kidney DMSA scan to confirm or exclude the presence of true renal involvement. These patients were well appearing and showed no pathological biomarker for SBI, no matter the screening method used. In a study of infants 2 to 24 months of age, 0.7% of afebrile girls had 3 successive urine cultures with 10^5^ CFUs per mL of a single uropathogen. Although difficult to distinguish, asymptomatic bacteriuria can be easily confused with true UTI in a febrile infant [19]. Moreover, studies assessing diagnosis of culture-confirmed UTIs with 99mTc-DMSA have demonstrated that as many as 30% febrile patients with UTIs do not show any renal involvement and therefore actually suffer from cystitis [20]. All patients with febrile UTIs were classified as SBIs because we considered the actual clinical diagnostic criteria for UTI. These patients could in fact represent the febrile proportion of children suffering from cystitis rather than true pyelonephritis. However, considering the pathological urine dipstick result, these children would have probably been prescribed antibiotics anyway for a suspected UTI. A Lab-score ≥3 was not able to detect a suspected *methicillin-sensitive Staphylococcus aureus* occult bacteremia in a 34 days old infant presenting with fever for 15 hours, CRP 27 mg/L, PCT 0.19 ng/mL, WBC count 28’600/mm^3^ and band count 860/mm^3^. The overall estimated annual incidence of blood stream infections in children aged 0–17 years is between 0.4/1000 (0.12/1000 in children without any comorbidity) and 0.7% in children 3 to 36 months of age [21]–[23]. Invasive *Staphylococcus aureus* infections are frequently encountered as nosocomial infections and are associated with selected risk factors such as age at the extreme spectrum of life, immunodeficiency, hemodialysis or peritoneal dialysis and the presence of intravenous catheters. This patient had no risk factor other than age, fever was moderate (38.4°C maximum) and except an elevated WBC count, all other SBI biomarkers remained negative. Fever regressed and did not recur after the first dose of ceftriaxone, which is not the first-line antibiotic in the treatment of MSSA. Although we concluded the diagnosis of true MSSA bacteremia for the purpose of the study, we nevertheless also considered the possibility of a potential contamination of the blood culture specimen by a MSSA strain. Finally, the last patient showing a negative Lab-score but a confirmed SBI presented *Escherichia coli* and enterovirus coinfection meningitis. This 70 day-old infant presented with an 8 hours-fever and showed the following biomarkers: CRP <10 mg/L, PCT 0.14 ng/mL, WBC count 25’200/mm^3^ and band count 1’510/mm^3^. Although rare, concurrent viral and bacterial meningitis can occur, with an incidence rate of 1.3–2.8% in patients suffering from meningitis [24]. In conclusion, a Lab-score ≥3 could not detect 10 cases of SBI, but the majority of them suffered from febrile UTIs with no certainty concerning renal involvement and 2 of them represented atypical clinical SBI cases. No single marker has already been shown 100% sensitive. A clinical follow-up is therefore essential to detect misdiagnosed cases, for patients with no diagnostic marker for SBI but persistent fever.

Several reasons can be raised regarding low adherence to the Lab-score recommendations. First, low adherence to guidelines has already been described by several authors [25]–[27]. Not surprisingly, the clinician frequently considers increased confidence in his own clinical feeling rather than in any diagnostic tool, which raises the question of translational medicine and bench- to-bedside considerations. Not only Lab-score recommendations, but also classical guidelines concerning FWS management are poorly observed. Indeed, now that the incidence of SBI has dropped following widespread pneumococcal vaccination [28], [29], a more conservative approach of well appearing infants or young children with FWS is frequently adopted [2], [30]–[32]. A recent study observed a decrease in ordering a complete blood count between 2004 and 2009 [2]. This tendency should be even more striking in the post PCV-13 era, with the increase in PCV-7 vaccination coverage and hence with decline in invasive pneumococcal disease and bacteremia. Second, the management of a patient requires a thorough appreciation of benefits and risks, and because values and preferences differ, clinical decision making will vary at an individual point of view [33]. Last, although recommendations concerning the Lab-score application had previously been published, residents were not routinely trained in using it before the study. Low confidence in this new tool could have resulted in low adherence rate to the related recommendations. A targeted educational intervention may have improved adhesion practices to the Lab-score. However, newer diagnostic strategies using either a more sequential approach to accurately identify children at risk for SBI (general appearance, then age and urinalysis result, and finally blood biomarkers), or the analysis of a continuous Lab-score spectrum rather than a defined cut-off are being evaluated and should be considered [34].

Our second objective was to test the accuracy of the Lab-score prospectively. The results of the diagnostic characteristics on the overall cohort showed an excellent area under the curve for the Lab-score (0.911), higher than any other single marker. The Lab-score showed both excellent positive and negative likelihood ratios (respectively 6.68 and 0.17). These results are consistent with the findings in previous retrospective studies evaluating the Lab-score as a diagnostic tool for SBI detection, both for ruling in and ruling out SBI [15], [17], [35].

Our study has limitations. First, potential recruitment bias may have occurred, since children with FWS were recruited at presentation to a tertiary care center only and do not represent the overall population of children with FWS (some of whom are primarily assessed by the primary care practitioner). Less severe infections and thus lower SBI prevalence are encountered in primary care settings, thus reducing the positive and negative predictive values of the Lab-score but having no impact on the sensitivity or specificity of the test. Second, our study as well as most of the previous ones has been conducted in a single center or a small group of tertiary care centers. Reproducibility should therefore be tested on larger multicenter studies. Another limitation to the study is that only 24.5% of children aged 7 days until 3 months of age presenting to the PED with FWS were enrolled in the study, either because inadvertent omission, lack of time to enroll patients and physician unwillingness to proceed to an extensive diagnostic testing in well-appearing patients thus inducing an inclusion bias. Indeed, the selection of a greater number of severe cases artificially raises the SBI incidence compared to the incidence found in the overall population of febrile children.

Another potential limitation to the study is the method used for fever measurement. Although the most reliable and precise method is rectal thermometry, the need for less invasive methods for temperature assessment made us define fever as presence of central body temperature equal to or greater than 38.0°C (100.4°F), measured either by rectal, axillary or transtympanic thermometers, either by parents or caregivers or by the nurse in charge of the patient at the time of presentation. These alternative methods could lack sensitivity or specificity concerning fever detection. Moreover, although nurses are well trained in using these devices, parents may lack experience and false temperature values may therefore be reported.

## Conclusions

Although the Lab-score showed identical sensitivity but better specificity than the combination of WBC count, band count and CRP regarding SBI detection, no difference regarding both antibiotic treatment rate and admission rate was observed when using the Lab-score compared to WBC and band counts and CRP determination, mostly because of lack of adherence to the Lab-score guidelines. Adherence to the commonly admitted recommendations based on WBC and band counts and CRP was also low. However, if the Lab-score had been strictly followed, a safe reduction of 26.5% antibiotic treatments would have been encountered. Medical education needs to be reinforced in order to observe rather than treat low-risk well-appearing children with FWS. New consensus guidelines focusing on FWS management need to be developed, in the light of the new epidemiology of SBI. Finally, the impact of the Lab-score on the antibiotic prescription and hospital referral rates should also be evaluated in primary care settings.

## Supporting Information

S1 Checklist **CONSORT 2010 Checklist.**
(DOC)

S1 Protocol **Full trial protocol (English).**
(DOC)

S2 Protocol **Full trial protocol (French).**
(DOC)
