# Supplementary material for: Impact of the Lab-Score on Antibiotic Prescription Rate in Children with Fever without Source: A Randomized Controlled Trial
Source: PLoS One. 2014 Dec 11;9(12):e115061. doi: 10.1371/journal.pone.0115061 (PMC4263728; doi:10.1371/journal.pone.0115061)
Supplement: S1 Protocol — Full trial protocol (English). (DOC) [file pone.0115061.s002.doc]

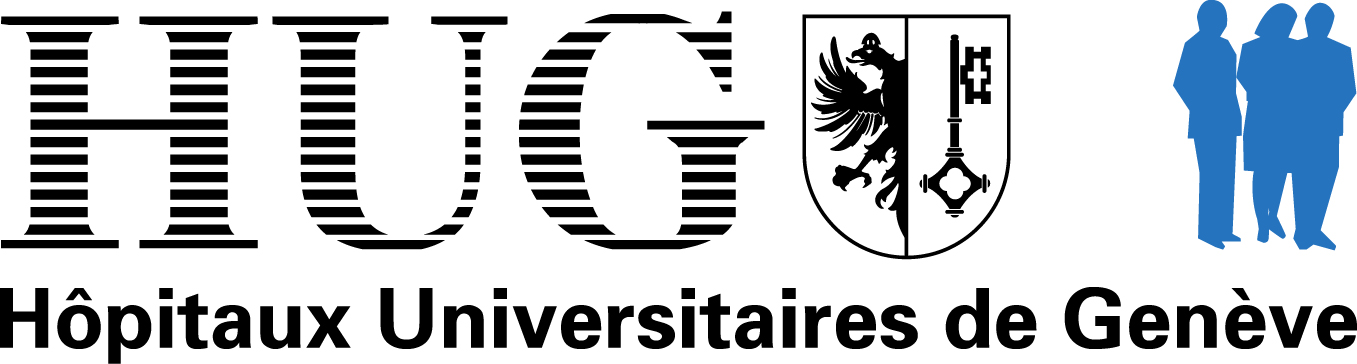
 STUDY PROTOCOL

**Utility of a biological score in decreasing antibiotic prescription rate in children from 7 days to 36 months old with fever without a source?**

Investigator : Dr Laurence LACROIX, M.D.

Pediatric Emergency Department

Child and Adolescent Medicine

Geneva University Hospital (HUG)

Avenue de la Roseraie 47

CH-1211 Geneva 14

Phone: +41 22 372 50 82

[laurence.lacroix@hcuge.ch](mailto:laurence.lacroix@hcuge.ch)

Co-investigators:

Prof Alain GERVAIX, M.D.

Head of the Pediatric Emergency Department, Pediatric Emergency Department, Child and Adolescent Medicine, Geneva University Hospital (HUG)

[alain.gervaix@hcuge.ch](mailto:alain.gervaix@hcuge.ch)

Dresse Annick GALETTO, M.D., Pediatric Emergency Department, Child and Adolescent Medicine, Geneva University Hospital (HUG)

[annick.galetto@hcuge.ch](mailto:annick.galetto@hcuge.ch)

Dr Sergio MANZANO, M.D., Pediatric Emergency Department, Child and Adolescent Medicine, Geneva University Hospital (HUG)

[sergio.manzano@hcuge.ch](mailto:sergio.manzano@hcuge.ch)

**Summary**

Fever without source (FWS) is a frequent reason for presentation to the Pediatric Emergency Service (PED). However, clinical examination and ancillary exams analyzed independently are not accurate enough to adequately detect serious bacterial infections (SBI) in children. This diagnostic challenge often leads to overprescription of antibiotics and thus increases both bacterial resistance and health costs.

The determination of a simple biological score called Lab-score, has been recently described. Based on the combined determination of procalcitonin (PCT), C-reactive protein (CRP) and presence of leukocyturia or nitrates on the urinary dipstick, the Lab-score has shown excellent diagnostic characteristics for SBI detection in children with fever without source on retrospective cohorts: 94% sensitivity and 81% specificity. Prospective application of the Lab-score should reduce unnecessary antibiotic prescription.

Our study aims at comparing antibiotic prescription rates in children with FWS aged 7 days up to 36 months of age presenting to the PED of Geneva University Hospital, Switzerland, after randomization into 2 groups: a group managed according to Lab-score determination (Lab-score group) and the other managed according to the results of commonly used biomarkers (control group): white blood cell count (WBC), band count, and CRP. Recruitment will occur from 01.09.2010 until 30.06.2013.

Demographical variables, incidence of SBI and of supposed or confirmed viral infections, antibiotic prescription rate and admission rate will be analyzed. A prospective validation of the Lab-score and of its diagnostic characteristics will then be possible, including the overall cohort.

We hope to encounter a 20% reduction antibiotic prescription rate in the Lab-score group compared to the control group, while maintaining an optimal security for the detection of SBI in children with FWS. A reduction in antibiotic prescription rate will certainly lead to benefic effects both on antibiotic resistance and on health costs.

**I. Introduction**

Fever is defined by elevated central core temperature ≥ 38.0 °C. It represents one of the most frequent reasons for presentation to the PED.

However, no source of infection will be found in 20% children after a detailed history and a thorough clinical examination[[1]](#endnote-2)ˉ[[2]](#endnote-3). Although the majority of these episodes represent viral infections, children aged less than 36 months are more at risk for occurrence of severe bacterial infection. These children need rapid antibiotic treatment since complications from untreated SBI can sometimes be devastating or lethal. SBI include sepsis, occult bacteremia, bacterial meningitis, febrile urinary tract infection (UTI), pneumonia, bacterial enteritis, osteomyelitis and septic arthritis.

Commonly used biomarkers show moderately accurate diagnostic characteristics for SBI detection, thus leading to frequent overprescription of antibiotics in children with FWS. It is nevertheless crucial to limit unnecessary antibiotic treatments in order reduce the increasing occurrence of bacterial resistance, potential complications associated with their use (drug allergy symptoms, diarrhea,…), to prevent from unnecessary hospital admissions associated with the need for parenteral antibiotic administration, and finally to reduce health costs.

**II. Literature review**

Fever without a source is a frequent diagnostic challenge in children presenting to the Pediatric Emergency Department, due to the difficulty in detecting SBI necessitating rapid adequate therapeutical management among a majority of viral or focal bacterial infections. Indeed, clinical signs analyzed independently are bad predictors for SBI[[3]](#endnote-4). Many tools have been described to increase the detection of SBI. The combined determination of the clinical score developed by Mc Carthy et al. (figure 1) together with WBC count is frequently used in clinical practice. However, these methods lack specificity and hence favor antibiotic prescription.

**Figure 1. Yale observation scale (from Pediatrics 1982;70:802-0)**

More recently, CRP and PCT determination have shown greater accuracy for SBI detection[[4]](#endnote-5)ˉ[[5]](#endnote-6)ˉ[[6]](#endnote-7).

CRP is an acute phase protein which is released by the liver in response to any inflammatory process or tissue damage. Its sensitivity and specificity are both questionable since low CRP values can be found despite underlying bacterial infectious processes and high CRP values may be detected during a viral infection. Moreover, CRP values begin to rise 12 hours only after the occurrence of fever, and a steady state is encountered at 20-72 hours.

PCT, the precursor of calcitonin, is produced by mainly cells throughout the body in case of bacterial infection. PCT blood levels already become detectable 2 hours after endotoxin administration on human experiments, and peaks at 6-8 hours[[7]](#endnote-8)ˉ[[8]](#endnote-9)ˉ[[9]](#endnote-10), with a steady state after 12 hours[[10]](#endnote-11)ˉ[[11]](#endnote-12) (figure 2). However, CRP and PCT can show serum peak crossovers when analyzed independently, thus inducing excessive antibiotic prescription.


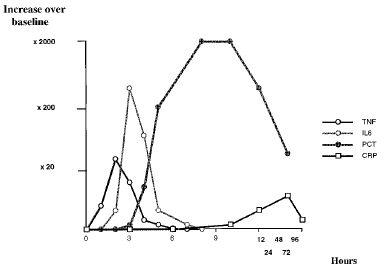


**Figure 2. Evolution of various infectious biomarker blood levels following endotoxin injection in healthy human volonteers**

*(adapted from J Clin Endocrinol Metabol, 1994, 79: 1605-8)*

A recently described biological score named Lab-score has been derived retrospectively from the variables the more strongly associated to SBI in a cohort of children with FWS aged 7 days until 36 months old. The Lab-score is based on the combined determination of CRP and PCT values and results of a urinary dipstick (figure 3).

| **Test** | **PCT** (**ng/mL)** | | | **CRP** (**mg/L)** | | | **Urinary dipstick** | |
| --- | --- | --- | --- | --- | --- | --- | --- | --- |
| **Value** | <0.5 | 0.5-1.99 | ≥ 2 | < 40 | 40-99 | ≥ 100 | negative | positive***** |
| **Points** | 0 | 2 | 4 | 0 | 2 | 4 | 0 | 1 |

**Figure 3. Lab-score calculation**

*****positive leukocyte esterase and/ or positive nitrate

*(adapted from Lacour, A.G., S.A. Zamora, and A. Gervaix, A score identifying serious bacterial infections in children with fever without source. Pediatr Infect Dis J, 2008.* ***27****(7): p. 654-)*

The Lab-score can be done easily in ambulatory settings with the use of POCTs (point of care tests), necessitating only an urine sample and a capillary or venous blood sample. In children less than 3 months old, 100 % sensitivity has been demonstrated, showing an excellent accuracy in detecting SBI and a 91% specificity, theoretically permitting a reduction in unnecessary antibiotics. On the entire cohort of children 7 days until 36 months old, sensitivity remained excellent (94%) and specificity very good (80%). Post test probability for SBI was 3% for a Lab-score value < 3 and 63% for a Lab-score value ≥3 in the studied population[[12]](#endnote-13).

The Lab-score has also been tested on an external population. Results remained excellent. (article recently submitted to the *Pediatric Infectious Diseases Journal*).

To our knowledge, there is however no prospective validation of the Lab-score.

**III. Objectives**

It is now commonly admitted that PCT is a precocious marker for bacterial infections. There is, to our knowledge, no study concerning the impact of a biological score including PCT determination on antibiotic prescription rates in children with FWS.

Research question: Does application of a biological score including urinary dipstick, CRP and PCT testing reduce antibiotic prescription rate in children 7 days until 36 months old with FWS at PED presentation?

**1. Main objective**

Our main objective is to compare **antibiotic prescription rates** between 2 groups of children 7 days until 36 months old with FWS at PED presentation: the first group managed according to the Lab-score value (Lab-score group) and the other group managed according to the results of commonly used biomarkers (control group), namely white blood cell count (WBC), band count, and CRP.

**2. Secondary objectives**

- To prospectively validate the diagnostic characteristics of the Lab-score to detect SBI in children with FWS: sensitivity, specificity, positive predictive value, negative predictive value of the Lab-score.
- To determine SBI rates, and the corresponding final diagnosis
- To compare treatment guideline adherence in both groups
- To compare hospitalization rates in both groups

**IV. Methods**

**1. Design**

We will perform a randomized controlled study in the Pediatric Emergency Department of Geneva University Hospital (HUG), Switzerland.

After parental written informed consent is obtained, assignment to either group will be achieved using chronologically numbered sealed envelopes containing indications either for the Lab-score group or for the control group. Randomization will be achieved through an Excel-generated random numbers table.

Patients in the **control group** will be managed according to commonly admitted biomarkers:

- WBC count
- Band count
- CRP
- Urinary dipstick (leukocytes or nitrates)
- Urine culture
- Blood culture
- Depending on clinical examination, with decision to perform left to the physician in charge of the patient: lumbar puncture, chest X-ray, or any other diagnostic exam aiming at narrowing the diagnosis.
- A supplemental 50 μl minimum serum sample will be frozen in order to analyze PCT later (thus Lab-score will not be performed when assessing the patient and PCT determination will NOT influence the decision to prescribe antibiotics).

Based on standard guidelines, indications whether antibiotics are recommended are provided in the data set for each patient, i.e. antibiotics recommended:

- If any abnormal biological result permits the diagnosis of a localized SBI (urinary tract infection, meningitis, pneumonia)
- If WBC count > 15’000 /mm³, or if band count > 1'500/mm³, or if CRP ≥40 mg/L
- In case the patient shows a toxic appearance

Patients in the **Lab-score group** will be managed according to the Lab-score value, and hence will undergo determination of:

- PCT (50 μl serum)
- CRP
- Urinary dipstick (leukocytes or nitrates)
- Urine culture
- Blood culture
- Depending on clinical examination, with decision to perform left to the physician in charge of the patient: lumbar puncture, chest X-ray, or any other diagnostic exam aiming at narrowing the diagnosis.
- WBC with differential: these results will be validated only 12 hours later, hence not influencing the decision to treat the patient with antibiotics.

Based on the Lab-score guidelines, indications whether antibiotics are recommended are provided in the data set for each patient, i.e. antibiotics recommended:

- If any abnormal biological result permits the diagnosis of a localized SBI (urinary tract infection, meningitis, pneumonia)
- If the Lab-score value is ≥ 3
- In case the patient shows a toxic appearance

After a 48- to 72-hour delay, a telephone follow-up will be carried out by one of the study investigators to assess the evolution of the clinical condition in the affected child, focusing on symptoms or signs that could have appeared in the interval from presentation. When the final diagnosis remained uncertain or when the fever was still present at the time of the initial phone follow-up, a free medical visit was offered to parents and further follow-up calls or visits were organized until definitive resolution of the fever episode for more than 24 hours. This follow-up will determine:

- Fever duration and evolution of the child’s condition
- New symptoms or signs aiming at precising the final diagnosis
- If any antibiotic treatment has been prescribed secondarily
- Need for hospitalization of the affected child.

Data will be recorded after anonymisation in a Microsoft Excel Database and then analyzed under PASW.

**2. Sample size calculation**

In the retrospective study describing the Lab-score derivation (derivation and application set), a 30% difference antibiotic prescription rate would have been encountered if the Lab-score had been strictly applied compared to the hypothetical treatment rate if recommendations based on commonly admitted biomarkers had been observed.

We aimed at showing a 20% reduction antibiotic prescription rate.

Sample size calculation concluded that 97 patients per group were necessary to show a 20% reduction antibiotic prescription rate with α= 0.05 and power 1-β=0.80. Taking into account the possibility of lost to follow-up patients and missing or incomplete results, we will consider including 140 patients in each group.

**3. Population**

The target population is the population of children aged 7 days until 36 months old presenting to the PED of Geneva University Hospital (HUG), Switzerland.

**4. Inclusion and exclusion criteria**

- **Inclusion criteria**
  - children 7 days until 36 months old
  - fever with central temperature ≥ 38.0°C (≥100.4°F)
  - no source of infection found after a detailed history and a thorough clinical examination
- **Exclusion criteria**
  - fever lasting > 7 days
  - age < 7 days or ≥ 36 months
  - antibiotics received in the previous 48 hours
  - immunodeficiency

**5. Description des méthodes de mesure**

- **Primary outcomes**
  - **In both groups** :
    - Antibiotic prescription rate
      - DCI name of the substance
      - Duration of antibiotic treatment prescribed
      - Prescribed dosage
    - Toxic appearance (defined as lethargy, poor peripheral perfusion, cyanosis, hypo- or hyperventilation)[[13]](#endnote-14)
    - CRP
    - Urinary dipstick
    - Urine culture
    - Blood culture
    - Depending on clinical examination :
      - Lumbar puncture
      - Chest X-ray
      - Rapid antigen testing…
  - **In the control group** :
    - WBC count with differential
    - 50 µl minimum frozen serum (for PCT determination)
  - **In the Lab-score group** :
    - PCT determination permitting calculation of the Lab-score value
    - WBC count with differential which results will be validated only 12 hours later, hence not influencing the decision to treat the patient with antibiotics.
- **Secondary outcomes**
  - Demographical data : age, gender, duration of fever before presentation, maximal measured temperature
  - FWS prevalence
  - SBI prevalence with underlying diagnosis
  - Adherence to treatment guidelines

**V. Statistical analysis project**

After anonymisation, data will be recorded using Microsoft Excel Database and then analyzed under PASW. The adequacy of randomization will be tested by comparing both groups. Normally distributed data will be expressed as mean ± standard deviation (SD), non-normally distributed data as median and interquartile range (IQR) and categorical data as percentages. Normally distributed data will be compared using independent-samples *t* test and non-normally distributed data using Mann–Whitney *U* test. Categorical data will be compared using χ2 test. We will consider parameters displaying p-values < 0.05 as statistically significant.

The diagnostic performance of the Lab-score and other laboratory markers will be analyzed using a receiver operating characteristic analysis. Sensitivity, specificity, positive and negative predictive values and likelihood ratios (LRs) at Lab-score ≥3 cutoff point will be calculated and reported with a 95% confidence interval (CI).

**VI. Possible bias and confounding variables**

Although inhomogeneous, the population of children presenting to a tertiary care center represents the most severely ill patients. Overestimation of SBI prevalence in such a setting may occur, thus influencing the positive predictive value of the Lab-score as well as potentially emphasizing the observed reduction in antibiotic prescription rate compared to that observed in primary care settings.

Moreover, the study will take place in a single academic center, introducing potential selection bias related to the presence of certain epidemic variables that could be absent in a different center.

Last, it is possible for the physician in charge of the patient to partially calculate the Lab-score value with CRP and urinary dipstick results only, even in the absence of PCT. The team in charge of the patient could therefore be able to apply recommendations of Lab-score in case the corresponding value is already elevated, introducing a confounding factor. However, the decision upon antibiotic treatment should follow the recommendations.

**VII. Expected results**

We encounter to observe a significant reduction (20% minimum difference) in antibiotic prescription rate in the group using the Lab-score compared to the control group. Moreover, because of the decreased need for parenteral antibiotics, we expect to observe a reduction in hospital admission rate.

Finally, we hope that following the Lab-score prospectively will show a good accuracy for the detection of SBI with an excellent security for infants and small children suffering from FWS.

VIII. Budget

| **EXAM** | **FUNDING** | **REMARKS** |
| --- | --- | --- |
| WBC count with differential  CRP  Blood culture  Urinary dipstick  Urine culture  ± Chest X-ray  ± Lumbar puncture  ± Rapid antigen testing | Patient insurance | Routine work-up for any patient suffering from FWS |
| PCT | bioMérieux | Loan of a Mini-Vidas and corresponding reagents |
| Data management and statistical analysis | 15'000 CHF funding from bioMérieux |  |

A PED consultant will be responsible for the study management. 3 co-investigators will perform the follow-up calls. A research nurse will help for the data management.

**IX. Calendar**

Submission of the project to the Institutional Ethics Committee: September 2009

Patient recruitment: August 2010- June 2013

Data analysis: July-October 2013

Writing of the article: November 2013-February 2014

**X. Conclusion**

FWS in children 7 days until 36 months old may mask an underlying SBI necessitating prompt recognition and adequate antibiotic treatment prescription. This frequent diagnostic challenge and hence induced overprescription of antibiotics prompts the need for a simple, rapid and efficient diagnostic tool in these patients. Using the sensitive and specific Lab-score should lead to a reduction in antibiotic prescription rate with an excellent security in affected infants and small children.

**XI. Bibliography**

1. Lee GM, Harper MB. Risk of bacteremia for febrile young children in the post**-***Haemophilus influenzae* type b era. *Arch Pediatr Adolesc Med.*1998;152:624–628 [↑](#endnote-ref-2)
2. Soman M. Characteristics and management of febrile young children seen in a university family practice. *J Fam Pract*. 1985;21:117–122 [↑](#endnote-ref-3)
3. Teach SJ, Fleisher GR, Occult Bacteremia Study Group. Efficacy of an observation scale in detecting bacteremia in febrile children three to thirty-six months of age, treated as outpatients. *J Pediatr*. 1995;126:877–881 [↑](#endnote-ref-4)
4. Van Rossum A, Wulkan R, Oudesluys-Murphy A. Procalcitonin as an early marker of infection in neonates and children. *The Lancet Infectious Diseases* 2004;4(10):620-630 [↑](#endnote-ref-5)
5. Hsiao AL, Baker MD. Fever in the new millennium: a review of recent studies of markers of serious bacterial infections in febrile children. *Curr Opin Pediatr.* 2005; 17:56-61 [↑](#endnote-ref-6)
6. Fernandez Lopez A, Cubells C, Garcia JJ, Pou J, The Spanish Society of Pediatric Emergencies. Procalcitonin in pediatric emergency departments for the early diagnosis of invasive bacterial infections in febrile infants: results of a multicenter study and utility of a rapid qualitative test fort this marker. *Pediatr Infect Dis J.* 2003;22:895-903 [↑](#endnote-ref-7)
7. Brunkhorst F, Heinz U, Forycki Z. Kinetics of procalcitonin in iatrogenic sepsis. *Intensive Care Med.* 1998;24:888-892 [↑](#endnote-ref-8)
8. Dandona P, Nix D, Wilson MF, Aljada A, Love J, Assicot M, Bohuon C. Procalcitonin increase after endotoxin injection in normal subjects. *J Clin Endocrinol Metab.* 1994;79:1605-1608. [↑](#endnote-ref-9)
9. Pulliam PN, Attia MW, Cronan KM. C-Reactive protein in febrile children 1 to 36 months of age with clinically undetectable serious bacterial infection. *Pediatrics.* 2001;108:1275-1279 [↑](#endnote-ref-10)
10. Putto A, Ruuskanen O, Meurman O, et al. C-reactive protein in the evaluation of febrile illness. *Arch Dis Childhood*. 1986;61:24–29 [↑](#endnote-ref-11)
11. Peltola H, Jaakkola M. C-reactive protein in early detection of bacteremic versus viral infections in imunocompetent and compromised children. *J Pediatr*. 1988;113:641–646 [↑](#endnote-ref-12)
12. Galetto Lacour A, Zamora SA, Gervaix A. A score identifying serious bacterial infections in children with fever without source. *Pediatr Infect Dis J*. 2008; 27: 654-656. Biological score to identify serious bacterial infections in children 25th European for Paediatric infectious diseases, 2007, Porto, Portugal. Poster #145 [↑](#endnote-ref-13)
13. McCracken G, Powell K, Baraff L, et al. Practice guideline for the management of infants and children 0 to 36 months of age with fever without source. *Pediatrics* 1993;92;1-12 [↑](#endnote-ref-14)
