# Supplementary material for: Impact of the Lab-Score on Antibiotic Prescription Rate in Children with Fever without Source: A Randomized Controlled Trial
Source: PLoS One. 2014 Dec 11;9(12):e115061. doi: 10.1371/journal.pone.0115061 (PMC4263728; doi:10.1371/journal.pone.0115061)
Supplement: S2 Protocol — Full trial protocol (French). (DOC) [file pone.0115061.s003.doc]

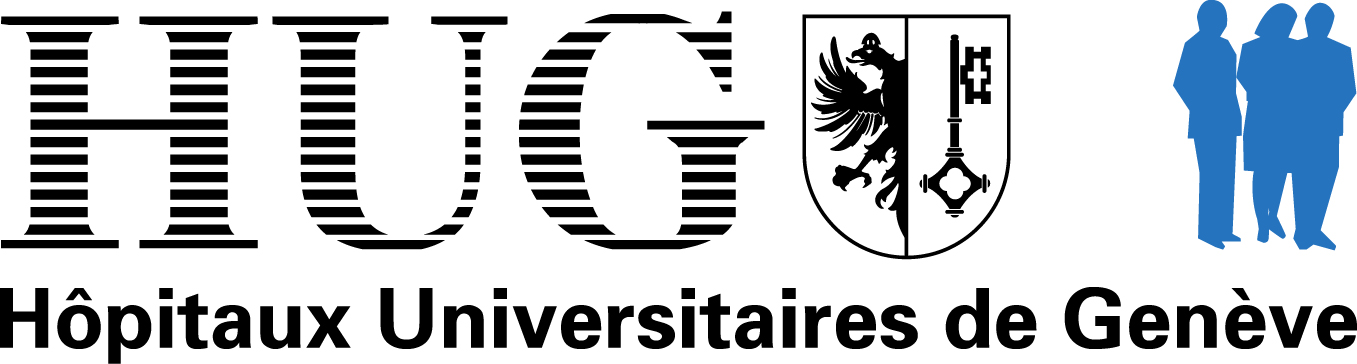
 TRIAL STUDY PROTOCOL

**L’utilisation d’un score biologique dans l’état fébrile sans foyer chez l’enfant de 7 jours à 36 mois réduit-elle la consommation d’antibiotiques ?**

Investigateur principal : Dr Laurence LACROIX

Médecin cheffe adjointe

Service d’Accueil et d’Urgences Pédiatriques

Département de l’Enfant et de l’Adolescent

Hôpitaux Universitaires de Genève HUG

Avenue de la Roseraie 47

CH-1211 Genève 14

Téléphone: +41 22 372 50 82

[laurence.lacroix@hcuge.ch](mailto:laurence.lacroix@hcuge.ch)

Co-investigateurs:

Prof Alain GERVAIX, médecin chef de service, Service d’Accueil et d’Urgences Pédiatriques, Département de l’Enfant et de l’Adolescent, Hôpital des Enfants, HUG ; [alain.gervaix@hcuge.ch](mailto:alain.gervaix@hcuge.ch)

Dresse Annick GALETTO, médecin adjointe, Service d’Accueil et d’Urgences Pédiatriques, Département de l’Enfant et de l’Adolescent, Hôpital des Enfants, HUG ; [annick.galetto@hcuge.ch](mailto:annick.galetto@hcuge.ch)

Dr Sergio MANZANO, médecin chef de clinique, Service d’Accueil et d’Urgences Pédiatriques, Département de l’Enfant et de l’Adolescent, Hôpital des Enfants, HUG ; [sergio.manzano@hcuge.ch](mailto:sergio.manzano@hcuge.ch)

**Résumé**

L’état fébrile sans foyer représente un motif fréquent de consultation dans les services d’urgences pédiatriques. Cependant, tant la clinique que les examens de laboratoire actuellement à disposition appréciés de manière isolée se révèlent insuffisants dans la détection et la prise en charge rapide de l’infection bactérienne sévère, induisant une surconsommation d’antibiotiques. Cette dernière favorise le développement de résistances bactériennes et agit défavorablement sur les coûts de la santé.

La détermination d’un score biologique simple et rapide, dénommé Laboratory Score, a récemment été décrite. Basé sur les résultats d’un stix urinaire et des valeurs sanguines de procalcitonine et de protéine C- réactive, ce score a démontré une excellente sensibilité (94%) quant à la détection de l’infection bactérienne sévère, avec une très bonne spécificité (81%).

L’application de ce score à une population évaluée de manière prospective devrait donc permettre une réduction de la consommation d’antibiotiques.

Notre étude vise à comparer la consommation d’antibiotiques, après randomisation, d’un groupe d’enfants chez lesquels sera appliqué le score par rapport à un groupe contrôle. La population visée est celle des enfants âgés de 7 jours à 36 mois de vie, consultant le service d’urgences pédiatriques des Hôpitaux Universitaires de Genève pour un état fébrile sans foyer détecté après une anamnèse et un examen clinique minutieux. Le recrutement aura lieu du 1^er^ septembre 2010 au 30 juin 2013.

Après une analyse de fréquence des variables démographiques et de l’incidence des infections bactériennes sévères, infections bactériennes focales, et infections supposées virales, les résultats comparant le taux de prescription d’antibiotiques dans les 2 groupes seront analysés par un test de Chi Carré. Une validation secondaire du score sera enfin effectuée avec le calcul de la sensibilité, spécificité, valeur prédictive positive et négative du Laboratory Score.

Nous espérons pouvoir démontrer une réduction de 20% du taux de prescription d’antibiotiques dans le groupe appliquant le Laboratory Score tout en maintenant une sécurité diagnostique adéquate par rapport à la détection de l’infection bactérienne sévère. Une diminution de la consommation d’antibiotiques aura secondairement un effet bénéfique sur la réduction des résistances bactériennes et sur les coûts de la santé.

**I. Introduction**

L’état fébrile, défini par une température centrale supérieure à 38°C représente l’un des motifs de consultation les plus courants au sein des services d’urgences pédiatriques.

Après une anamnèse et un examen clinique minutieux, environ 20% des enfants n’auront pas de foyer clinique évident permettant d’expliquer la symptomatologie fébrile[[1]](#endnote-2)ˉ[[2]](#endnote-3). Bien que la majorité des infections soit d’origine virale, les enfants de moins de 36 mois sont néanmoins à risque de présenter une infection bactérienne sérieuse requérant une intervention thérapeutique rapide. Les infections bactériennes sérieuses comprennent principalement : la bactériémie occulte, la méningite, la pyélonéphrite, la pneumonie, l’arthrite septique et l’ostéomyélite.

Les tests utilisés en pratique courante ont une spécificité réduite quant à la détection de l’infection bactérienne sérieuse, induisant une surconsommation d’antibiotiques. Il est toutefois important de veiller à limiter l’utilisation de ces derniers afin de réduire le développement de résistances bactériennes, les complications associées à leur administration, de prévenir les hospitalisations non nécessaires pour antibiothérapie parentérale et finalement d’agir sur les coûts de la santé.

**II. Revue de la littérature**

L’état fébrile sans foyer représente un problème diagnostique fréquent, de part la difficulté à différencier une infection virale d’une infection bactérienne sérieuse nécessitant une intervention thérapeutique rapide. En effet, les signes cliniques sont de mauvais prédicteurs d’infection bactérienne sérieuse[[3]](#endnote-4). Différents outils ont été développés afin d’accroître la sensibilité de dépistage de l’infection bactérienne sérieuse. Parmi ceux-ci, le score clinique développé par Mc Carthy et al. (figure 1) associé aux valeurs biologiques de la formule sanguine complète sont fréquemment utilisés. Toutefois, ces méthodes restent peu spécifiques, induisant une surconsommation d’antibiotiques.

**Qualité des pleurs**Forts, tonalité normale Sanglots ouFaibles ou plaintifs

ou content, ne pleure pas gémissementsou tonalité aiguë

**Réaction à la stimu-**Content, ne pleure pas ou Pleurs intermittentsPleurs incessants ou

**lation des parents**pleure brièvement puis inconsolable

stoppe

**Etat d’éveil**Si éveillé reste éveillé, si Ferme yeux brièvementS’endort ou ne se

endormi et stimulé, se puis s’éveille ou éveillableréveille pas

réveille rapidement après stimulation prolongée

**Couleur**Rose Extrémités pâles ouPâle ou cyanosé ou acrocyanose marbré ou grisâtre

**Hydratation**Peau et yeux: sp. et Peau et yeux sp. et Signe du pli et

muqueuses humides muqueuses ± sèchesmuqueuses sèches

et/ou yeux enfoncés

**Contact social**Sourit ou alerte (≤2 mois) Sourire bref ou alerteAucun sourire, visage

peu de temps (≤2 mois)anxieux sans expression ou pas alerte

Observations**Normal Atteinte modéréeAtteinte sévère**

**1 35**

**Figure 1. Score clinique de Mc Carthy (Adapté de Pediatrics 1982;70:802-0)**

Plus récemment, la détermination des valeurs sanguines de protéine C-réactive (CRP) et de procalcitonine (PCT) a démontré une plus grande fiabilité diagnostique quant à la sensibilité pour la détection des infections bactériennes sérieuses[[4]](#endnote-5)ˉ[[5]](#endnote-6)ˉ[[6]](#endnote-7).

La CRP est une protéine de la phase aiguë, produite par le foie en réponse à un état inflammatoire ou à un dommage tissulaire. Sa spécificité et sa sensibilité restent cependant discutables puisqu’elle peut rester basse en cas d’infection bactérienne et augmenter en cas d’infection virale. De plus, la concentration de la CRP ne commence à augmenter que 12 heures après le début de l’épisode fébrile, et atteint plateau après 20-72 heures.

La PCT est un peptide précurseur de la calcitonine, qui est produit par de nombreux tissus en réponse à l’infection bactérienne. Sa concentration devient détectable au niveau sérique 2 heures après l’administration d’endotoxine expérimentale avec un pic de concentration maximale à 6-8 heures[[7]](#endnote-8)ˉ[[8]](#endnote-9)ˉ[[9]](#endnote-10), et un plateau atteint après environ 12 heures[[10]](#endnote-11)ˉ[[11]](#endnote-12) (figure 2). Toutefois, il existe un chevauchement des différentes variables lorsqu’elles sont appréciées de manière indépendante, conduisant là aussi à un taux de prescription d’antibiotiques élevé.


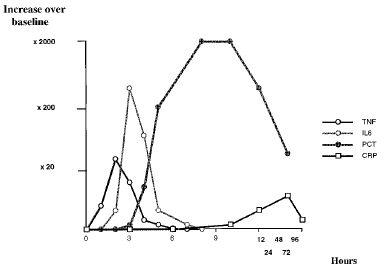


**Figure 2. Cinétique des différents marqueurs infectieux après injection d’endotoxine chez des volontaires sains** (J Clin Endocrinol Metabol, 1994, 79: 1605-8)

Une étude a récemment développé un score défini comme un index de risque d’infection bactérienne sérieuse chez des enfants de 7 jours à 36 mois consultant un service d’urgences pédiatriques pour un état fébrile sans foyer. Basé sur les valeurs de PCT, de CRP et du stix urinaire (figure 3), il a été développé après analyse multivariée de régression logistique sur les variables les plus fortement associées à l’infection bactérienne sérieuse en analyse univariée.

| Examen | **PCT** **ng/mL** | | | **CRP** **mg/L** | | | **Stix urinaire** | |
| --- | --- | --- | --- | --- | --- | --- | --- | --- |
| Valeur biologique | <0.5 | 0.5-1.99 | ≥ 2 | < 40 | 40-99 | ≥ 100 | négatif | positif* |
| Nombre de points | 0 | 2 | 4 | 0 | 2 | 4 | 0 | 1 |

Figure 3. Calcul du Laboratory Score *leucocyturie ou présence de nitrites

Ce score biologique, dénommé Laboratory Score, peut être effectué en pratique ambulatoire au moyen de tests rapides, après prise de sang capillaire. Chez l’enfant de moins de 3 mois, il a démontré une sensibilité de 100 % traduisant une sécurité adéquate quant à la détection de l’infection bactérienne sérieuse et une spécificité de 91%, permettant de limiter la prescription d’antibiotiques aux patients en ayant réellement besoin. Sur la population étudiée des enfants de 7 jours à 36 mois, la sensibilité restait excellente à 94% et la spécificité à 80%. La probabilité d’infection bactérienne sérieuse post-test est de 3% pour un score < 3 et de 63% pour un score ≥3 dans la population étudiée[[12]](#endnote-13).

Ce même score a également été validé sur une population externe, et ses résultats restent excellents (article soumis récemment par notre équipe au *Pediatric Infectious Diseases Journal*).

Il n’existe cependant pas, à notre connaissance, de validation de ce score sur une étude prospective.

**III. Objectifs**

Il est donc actuellement bien établi que la PCT est un des marqueurs précoces de l’infection bactérienne. Cependant, il n’existe à notre connaissance aucune étude ayant démontré une réduction de la consommation d’antibiotiques grâce à l’utilisation d’un score biologique incluant la PCT en routine quant à la détection des infections bactériennes sérieuses chez l’enfant présentant un état fébrile sans foyer.

Question de recherche : L’utilisation d’un score biologique basé sur les valeurs du stix urinaire, de la CRP et de la PCT réduit-il la consommation d’antibiotiques chez des enfants âgés de 7 jours à 36 mois, consultant un service d’urgences pédiatriques pour un état fébrile sans foyer ?

**1. Objectif principal**

Notre objectif est donc de **comparer la consommation d’antibiotiques** chez les enfants âgés de 7 jours à 36 mois consultant les urgences pédiatriques pour un état fébrile sans foyer dans 2 groupes distincts: l’un bénéficiant du schéma diagnostique de prise en charge au moyen des examens standards actuellement utilisés (FSC, CRP, stix urinaire), l’autre bénéficiant de l’application du score (Laboratory Score exprimé ci-dessus), après mesure de la CRP, de la PCT et des valeurs du stix urinaire.

**2. Objectifs secondaires**

Les objectifs secondaires de cette étude sont les suivants :

- Valider prospectivement que l’utilisation du Laboratory Score permet une prédiction de l’infection bactérienne sérieuse dans l’état fébrile sans foyer tout en maintenant une sécurité diagnostique adéquate. Calcul de sensibilité, spécificité, VPP, VPN du Laboratory Score
- Calculer le taux d’IBS, d’infection bactérienne focale et d’infection virale probable
- Comparer l’adhérence aux recommandations de traitement au sein des 2 groupes
- Comparer le taux d’hospitalisation

**IV. Méthodes**

**1. Design**

Il s’agit d’une étude prospective, randomisée, contrôlée, menée au sein du Service d’Accueil et d’Urgences Pédiatriques des Hôpitaux Universitaires de Genève (HUG), Suisse.

Après obtention d’un consentement écrit éclairé de la part des parents ou du responsable légal de l’enfant, les patients seront randomisés au moyen d’enveloppes scellées attribuant le patient à l’un des 2 groupes, de façon aléatoire.

Les patients inclus dans le **groupe « contrôle »** bénéficieront de la prise en charge classique en vigueur :

- FSC
- CRP
- Stix urinaire
- Culture d’urine
- Hémoculture
- Selon la clinique : ponction lombaire, radiographie du thorax, ou autre examen diagnostique supplémentaire lié au libre choix du médecin en charge du patient.
- Le seul prélèvement supplémentaire à la prise en charge standard est un échantillon de sérum de 50 μl au minimum, qui sera prélevé et congelé afin de pouvoir analyser la PCT a posteriori (dont le résultat a posteriori n’influencera donc pas ni le score ni la prise en charge)

Le choix de traiter ou non le patient par antibiotiques suivra les recommandations thérapeutiques actuelles, à savoir, pour le groupe « contrôle », prescription d’antibiotiques :

- en cas d’anomalie biologique permettant la mise en évidence secondaire d’un foyer (par exemple infection urinaire, méningite, pneumonie)
- en cas de leucocytose > 15’000 /mm³, ou déviation gauche (neutrophiles non segmentés > 1'500/mm³), ou CRP ≥40 mg/L
- en cas d’apparence toxique

Les patients inclus dans le **groupe « score»** subiront les analyses suivantes, permettant notamment le calcul du Laboratory Score :

- PCT (50 μl de sérum, seul prélèvement supplémentaire à la prise en charge standard)
- CRP
- Stix urinaire
- Culture d’urine
- Hémoculture
- Selon clinique : ponction lombaire, radiographie du thorax, ou autre examen diagnostique supplémentaire lié au libre choix du médecin en charge du patient.
- Une FSC sera effectuée au laboratoire, et les résultats seront tenus secrets et validés par le laboratoire seulement 12 heures plus tard.

Le choix d’administrer des antibiotiques à l’un des patients du groupe « score » suivra les guidelines établis, à savoir antibiothérapie préconisée :

- en cas d’anomalie biologique permettant la mise en évidence secondaire d’un foyer (par exemple infection urinaire, méningite, pneumonie)
- en cas de Laboratory Score ≥ 3
- en cas d’apparence toxique

Tous les patients seront contactés par téléphone à 48-72 heures par l’un des médecins de l’équipe des urgences. Ceux dont l’évolution n’est pas satisfaisante après l’un des 2 contacts téléphoniques seront convoqués aux HUG pour un contrôle clinique sans frais supplémentaire. Ce feed-back nous permettra d’obtenir :

- L’évolution de l’état fébrile (persistance ou non) et de l’état général de l’enfant
- L’apparition éventuelle de nouveaux symptômes et/ou signes cliniques, permettant de poser un éventuel diagnostic
- La prescription éventuelle d’antibiotiques dans l’intervalle par le médecin traitant
- L’hospitalisation éventuelle de l’enfant dans l’intervalle

Au terme de cette collecte, les données des patients seront anonymisées dans le logiciel d’analyse statistique, sous leur numéro d’inclusion dans l’étude.

**2. Taille d’échantillon**

Dans l’étude rétrospective ayant permis d’établir le Laboratory Score, on aurait observé une différence de taux de traitement antibiotique de 30% entre les 2 groupes (avec un taux de traitement moindre dans le groupe score par rapport au groupe standard) si les patients avaient été traités selon les résultats du score plutôt que selon l’attitude standard.

Ici, l’objectif visé est donc de pouvoir démontrer une réduction de ***20%*** du taux de prescription d’antibiotiques.

Pour un α= 0.05 et une puissance 1-β=0.80, le calcul de taille d’échantillon nécessaire est de 97 patients dans chaque groupe. Nous avons donc opté pour inclure 140 participants dans chaque groupe, prenant en compte la possibilité de patients perdus lors du suivi ou de résultats manquants ou incomplets.

**3. Population**

La population visée est celle des enfants âgés de 7 jours à 36 mois, consultant le Service d’Accueil et d’Urgences Pédiatriques des Hôpitaux Universitaires de Genève (HUG), Suisse.

**4. Critères d’inclusion et d’exclusion**

- **Critères d’inclusion**
  - Âge de 7 jours de vie à 36 mois
  - Etat fébrile avec température centrale > 38°C
  - Absence de foyer clinique mis en évidence après une anamnèse et un examen clinique minutieux
- **Critères d’exclusion**
  - État fébrile de durée > 7 jours
  - Âge < 7 jours ou > 36 mois
  - Antibiothérapie durant les 48 dernières heures
  - Immunodéficience

**5. Description des méthodes de mesure**

- **Mesures principales**
  - **Pour les 2 groupes** :
    - Taux de prescription d’antibiotiques
      - Nom DCI de l’antibiotique
      - durée de traitement prescrite
      - dosage prescrite
    - Apparence toxique (présentation clinique caractérisée par une léthargie, une mauvaise perfusion, une cyanose, et une hypo- ou hyperventilation)[[13]](#endnote-14)
    - CRP
    - stix urinaire
    - culture d’urine
    - hémoculture
    - Selon clinique :
      - ponction lombaire
      - radiographie du thorax
      - Tests antigéniques rapides…
  - **Groupe « contrôle»** :
    - FSC
    - Sérum congelé (analyse de la PCT a posteriori)
  - **Groupe « score »** :
    - PCT permettant le calcul du Laboratory Score
    - FSC dont les résultats ne seront validés par le laboratoire que 12 heures plus tard, non communiqués au médecin prenant en charge le patient
- **Mesures secondaires**
  - Mesures démographiques : âge, sexe, durée de l’état fébrile avant consultation, température maximale
  - Prévalence des cas d’état fébrile sans foyer
  - Taux d’infection bactérienne sérieuse, d’infection bactérienne focale et d’infection virale probable dans chacun des groupes
  - Adhérence aux recommandations de traitement

**V. Projet d’analyse statistique**

Les données seront entrées de façon anonymisée dans une base de données Microsoft Excel puis anlysées via SPSS.

Un calcul de fréquence sera effectué afin de vérifier une répartition équivalente dans les 2 groupes des variables démographiques recueillies. Les valeurs démontrant une distribution normale seront exprimées en valeur moyenne ± déviation standard (SD), les valeurs ne démontrant pas de distribution normale en médiane et écart interquartile (IQR) et les valeurs catégoriques en pourcentages. Les valeurs dont la distribution est normale seront comparées par test de *t* pour échantillons indépendants. Le valeurs dont la distribution n’est pas normale par un test *U* de Mann–Whitney. Les valeurs catégoriques seront comparées par un test de χ2.

Une différence statistiquement significative sera prise en compte lors de valeur p < 0.05.

On analysera également les fréquences relatives d’infection bactérienne sévère, d’infection bactérienne focale et d’infection supposée virale au sein des 2 groupes.

Par ailleurs, un calcul de la sensibilité, spécificité, valeur prédictive positive et négative du Laboratory Score sera également réalisée.

**VI. Evocation de possibles biais et confondants**

Bien qu’hétérogène, la population consultant un centre de référence hospitalier représente un sous-groupe de patients les plus malades, d’où une potentielle surestimation de la fréquence d’infection bactériennes sévères, de la validité du score, et donc de l’effet sur la consommation d’antibiotiques, par rapport à la pratique de cabinet.

Par ailleurs, il pourrait exister un biais lié à des facteurs épidémiques transitoires, induisant la présence d’agents infectieux potentiellement responsables de l’état fébrile sans foyer, qui ne serait pas reproductibles dans d’autres centres.

L’utilisation d’un score partiel dans le groupe standard (calculé de façon partielle après obtention de la CRP et du stix urinaire) peut être un confondant potentiel. Cependant, la décision de donner ou non l’antibiotique devrait suivre les recommandations.

**VII. Résultats attendus**

Nous nous attendons à observer une différence significative de consommation d’antibiotiques dans les 2 groupes, avec une réduction de la prescription d’environ 20% dans le groupe appliquant le score par rapport au groupe appliquant la prise en charge standard.

Par ailleurs, nous pensons également observer une légère diminution du taux d’hospitalisation.

Nous espérons que l’utilisation du Laboratory Score de manière prospective montrera de bons résultats quant à la sensibilité, spécificité et valeurs prédictives positive et négative.

VIII. Budget

| **EXAMEN** | **FINANCE MENT** | **REMARQUE** |
| --- | --- | --- |
| FSC  CRP  hémoculture  stix  culture d’urine  ± radiographie du thorax  ± ponction lombaire  ±test antigénique rapide | À charge du patient / assurance maladie | Examens de routine pour le diagnostic d’un état fébrile sans foyer. |
| PCT | bioMérieux | Matériel mis à disposition par bioMérieux |
| Analyse statistique | Aide de 15'000 CHF versée par bioMérieux (cf contrat annexé). | Aide à l’analyse biostatistique et au data management |

Un chef de clinique des urgences est responsable de l’étude et de son bon fonctionnement. Une infirmière de recherche et un assistant de recherche seront également disponibles afin de rappeler les parents et d’aider à la gestion de la base de données de l’étude. Les patients seront rappelés à 24 heures et à 48-72 heures et revus ou par l’un des internes et/ou chefs de clinique des urgences en cas de péjoration clinique, comme cela est déjà fréquemment le cas, sans facturation supplémentaire.

**IX. Calendrier**

Soumission du projet à la commission d’éthique des HUG : septembre 2009

Recrutement des patients: août 2010- juin 2013

Analyse des résultats : juillet-octobre 2013

Rédaction d’article : novembre 2013-février 2014

**X. Conclusions**

L’état fébrile sans foyer de l’enfant de 7 jours à 36 mois peut parfois être le signe d’une infection bactérienne sévère requérant une intervention thérapeutique rapide. De par sa fréquence et la surconsommation d’antibiotiques induite, la prise en charge de ces patients aux urgences nécessite le développement d’une méthode diagnostique simple, rapide et efficace quant à la détection d’une éventuelle infection bactérienne sévère sous-jacente. Par l’utilisation d’un score simple, facile à retenir, rapide d’utilisation, et plus spécificique quant à la détection de l’infection bactérienne sévère que les méthodes actuellement à disposition, nous espérons observer une diminution de la prescription d’antibiotiques, tout en maintenant une sécurité diagnostique adéquate.

**XI. Bibliographie**

1. Lee GM, Harper MB. Risk of bacteremia for febrile young children in the post**-***Haemophilus influenzae* type b era. *Arch Pediatr Adolesc Med.*1998;152:624–628 [↑](#endnote-ref-2)
2. Soman M. Characteristics and management of febrile young children seen in a university family practice. *J Fam Pract*. 1985;21:117–122 [↑](#endnote-ref-3)
3. Teach SJ, Fleisher GR, Occult Bacteremia Study Group. Efficacy of an observation scale in detecting bacteremia in febrile children three to thirty-six months of age, treated as outpatients. *J Pediatr*. 1995;126:877–881 [↑](#endnote-ref-4)
4. Van Rossum A, Wulkan R, Oudesluys-Murphy A. Procalcitonin as an early marker of infection in neonates and children. *The Lancet Infectious Diseases* 2004;4(10):620-630 [↑](#endnote-ref-5)
5. Hsiao AL, Baker MD. Fever in the new millennium: a review of recent studies of markers of serious bacterial infections in febrile children. *Curr Opin Pediatr.* 2005; 17:56-61 [↑](#endnote-ref-6)
6. Fernandez Lopez A, Cubells C, Garcia JJ, Pou J, The Spanish Society of Pediatric Emergencies. Procalcitonin in pediatric emergency departments for the early diagnosis of invasive bacterial infections in febrile infants: results of a multicenter study and utility of a rapid qualitative test fort this marker. *Pediatr Infect Dis J.* 2003;22:895-903 [↑](#endnote-ref-7)
7. Brunkhorst F, Heinz U, Forycki Z. Kinetics of procalcitonin in iatrogenic sepsis. *Intensive Care Med.* 1998;24:888-892 [↑](#endnote-ref-8)
8. Dandona P, Nix D, Wilson MF, Aljada A, Love J, Assicot M, Bohuon C. Procalcitonin increase after endotoxin injection in normal subjects. *J Clin Endocrinol Metab.* 1994;79:1605-1608. [↑](#endnote-ref-9)
9. Pulliam PN, Attia MW, Cronan KM. C-Reactive protein in febrile children 1 to 36 months of age with clinically undetectable serious bacterial infection. *Pediatrics.* 2001;108:1275-1279 [↑](#endnote-ref-10)
10. Putto A, Ruuskanen O, Meurman O, et al. C-reactive protein in the evaluation of febrile illness. *Arch Dis Childhood*. 1986;61:24–29 [↑](#endnote-ref-11)
11. Peltola H, Jaakkola M. C-reactive protein in early detection of bacteremic versus viral infections in imunocompetent and compromised children. *J Pediatr*. 1988;113:641–646 [↑](#endnote-ref-12)
12. Galetto Lacour A, Zamora SA, Gervaix A. A score identifying serious bacterial infections in children with fever without source. *Pediatr Infect Dis J*. 2008; 27: 654-656. Biological score to identify serious bacterial infections in children 25th European for Paediatric infectious diseases, 2007, Porto, Portugal. Poster #145 [↑](#endnote-ref-13)
13. McCracken G, Powell K, Baraff L, et al. Practice guideline for the management of infants and children 0 to 36 months of age with fever without source. *Pediatrics* 1993;92;1-12 [↑](#endnote-ref-14)
